# Forelimb muscle activity during level and progressive incline and decline walking in dogs and implications for rehabilitation

**DOI:** 10.3389/fvets.2025.1649009

**Published:** 2025-08-29

**Authors:** Rachel Mariël Cain, Kate Stanford, Marti Drum, Jim Richards, David Levine, Darryl Millis, Tena Ursini

**Affiliations:** ^1^Department of Small Animal Clinical Sciences, University of Tennessee College of Veterinary Medicine, Knoxville, TN, United States; ^2^Allied Health Research Unit, University of Lancashire, Lancashire, United Kingdom; ^3^Department of Physical Therapy, University of Tennessee at Chattanooga, Chattanooga, TN, United States; ^4^Department of Large Animal Clinical Sciences, College of Veterinary Medicine, Equine Performance and Rehabilitation Center, University of Tennessee, Knoxville, TN, United States

**Keywords:** electromyography, rehabilitation, supraspinatus, biceps brachii, triceps brachii, deltoideus, incline walk, decline walk

## Abstract

**Introduction:**

Shoulder pathologies are a common reason for presentation to veterinary sports medicine and rehabilitation practices. Currently there are no standardized rehabilitation protocols for shoulder injuries but controlled walking, either on flat ground or on inclines/declines, is recommended in nearly every recovery rehabilitation program. The objective of this study was to evaluate the peak and average muscle activity of commonly targeted forelimb muscle groups using fine-wire and surface electromyography (EMG) during treadmill walking at five treadmill positions. Our hypothesis was that the forelimb muscle activity would be significantly higher during decline walking than level walking and significantly lower during incline walking.

**Methods:**

Fine-wire and surface EMG of the supraspinatus, deltoideus, biceps brachii and lateral head of the triceps brachii muscles were performed during treadmill walking at 0, 5% incline, 10% incline, 5% decline, and 10% decline. The average and peak muscle enveloped EMG signals for 10 gait cycles were compared between the treadmill positions.

**Results:**

Significant main effects were seen during decline walking for average supraspinatus muscle activity (*p* < 0.001), and both average and peak deltoideus muscle activity (*p* = 0.021, *p* < 0.001) respectively. There were no significant differences for peak or average lateral triceps brachii or biceps brachii activity between treadmill positions.

**Conclusion:**

Decline walking significantly increases muscle activity in the supraspinatus and deltoideus muscles of dogs. This study provides new insights regarding the muscle activity of the thoracic limbs in dogs during various treadmill positions at the walk. The impact of incremental treadmill positions on the average supraspinatus EMG activity, along with increases in the average and peak EMG activity of the deltoideus muscle during decline walking should be considered when developing a therapeutic exercise plan in canine patients with shoulder injuries.

## 1 Introduction

Shoulder pathologies are increasingly recognized as causes of lameness and functional impairment, particularly in sporting and working dogs (1, 2). Owner-based surveys, observational, and retrospective studies have identified the shoulder as a common joint injured in agility, flyball, racing, canicross, and sled dogs (3–8). Consequently, canine sports medicine and rehabilitation practitioners frequently diagnose and treat shoulder conditions in companion, sporting and working dog patients.

Supraspinatus and bicipital tendinopathies are two commonly identified shoulder ailments of dogs, and both are often managed conservatively with rehabilitation (1–3). In almost every rehabilitation program, walking appears to be a valuable therapeutic exercise despite its simplicity (2, 9). Walking exercises on level ground, inclines, and declines are commonly used in canine rehabilitation for a wide variety of conditions such as hip dysplasia (10), stifle injuries (11, 12), biceps tenosynovitis (13), and neurological conditions such as intervertebral disc disease (14, 15). Performing rehabilitation exercises on treadmills may provide unique advantages during exercise including precise control over velocity and the amount of incline or decline. Treadmills also reduce external influences such as variable terrain or environmental factors and can also be used for gait analysis (16–18).

Walking on incline or decline surfaces involves adjustments of the head, trunk, and limbs and has been reported to alter muscle activity in dogs. Surface EMG of dogs walking on an inclined treadmill at 5% (19), and 7% (20) increased activity of the biceps femoris and middle gluteal muscles compared with level walking and decline walking (19, 20). Although there is some data on uphill and downhill walking, no comparisons of the incremental effects of incline and decline walking have been reported.

Most of the canine electromyography (EMG) studies during specific therapeutic exercises have assessed hindlimb muscle activity (17, 21–23). Studies examining EMG of the forelimbs are more limited. Garcia et al. (24) evaluated the muscle activity of the elbow flexor and extensor muscle groups in normal walking and trotting dogs using surface EMG to provide a reference for clinical evaluation and study of locomotor abnormalities. Janas et al. (25) evaluated the muscle activity of biceps brachii and lateral triceps muscles during walking, trotting, and selected therapeutic exercises and showed the mean and peak EMG amplitude of both muscles increased during walking and trotting compared to stance. In addition, Cullen et al. (26, 27) evaluated the electromyographic activity of the left biceps brachii, supraspinatus, infraspinatus, and triceps brachii muscles using fine-wire EMG while performing dynamic, highly specific agility-related tasks. However, there is a gap in the current literature on the forelimb muscle activity when considering early recovery exercises such as incline and decline treadmill walking. This EMG data may help in developing progressive therapeutic exercise programs for forelimb injuries, especially with the current prevalence of shoulder pathologies.

Inertial measurement units (IMUs) have been used as a convenient wearable method over marker based kinematic systems for human gait analysis, and several canine studies have evaluated and verified the use of IMUs for gait analysis in dogs. IMU technology seemingly provides accurate and reliable determination of stride parameters in dogs (28–31).

Objective and Hypothesis: The objective of this study was to evaluate the peak and average muscle activity of commonly targeted forelimb muscle groups using fine-wire and surface EMG during treadmill walking at five treadmill positions: 0, 5% incline, 10% incline, 5% decline, and 10% decline. We hypothesized that the forelimb muscle activity would be significantly greater during decline walking than level walking and significantly less during incline walking.

## 2 Materials and methods

All dogs had complete neurologic and orthopedic examinations performed by a board-certified sports medicine and rehabilitation veterinarian prior to enrollment; dogs were excluded if they showed any signs of visible lameness or pain upon palpation of the joints, spine, or skeletal muscles or if they had any gait abnormality at the walk or trot, posture abnormality, or any other orthopedic or neurologic conditions. To confirm that dogs had no lameness, kinetic data were obtained using a force platform (AMTI, Watertown, MA). Four valid trials for each side of the dog were obtained at a trot. Velocity and acceleration of the dog was maintained between 1.7 and 2.1 m/s and ±0.40 m/s^2^. Mean peak vertical force values were used to identify weight-bearing left–right asymmetry for each dog; dogs were excluded from the study if there was > 5% asymmetry between the forelimbs or hindlimbs. Eighteen dogs were screened and two were excluded due to lameness on the kinetic analysis and abnormal orthopedic examination and one dog was excluded due to abnormal orthopedic examination but had a normal kinetic analysis.

Twelve dogs were objectively and subjectively clinically sound and qualified for the study. This study was approved by the University of Tennessee Institutional Animal Care and Use Committee (protocol #3075) and was performed in accordance with AAALAC and USDA guidelines.

### 2.1 EMG and IMU sensors

We evaluated muscle activity bilaterally in the supraspinatus, biceps brachii, scapular portion of deltoideus, and lateral head of triceps brachii muscles. Muscle bellies were located by manual palpation. Large areas around the sensor placement were clipped and then shaved with a razor, and the skin was cleaned with chlorhexidine scrub and isopropyl alcohol to remove oil, dirt, and debris.

**Figure 1:**
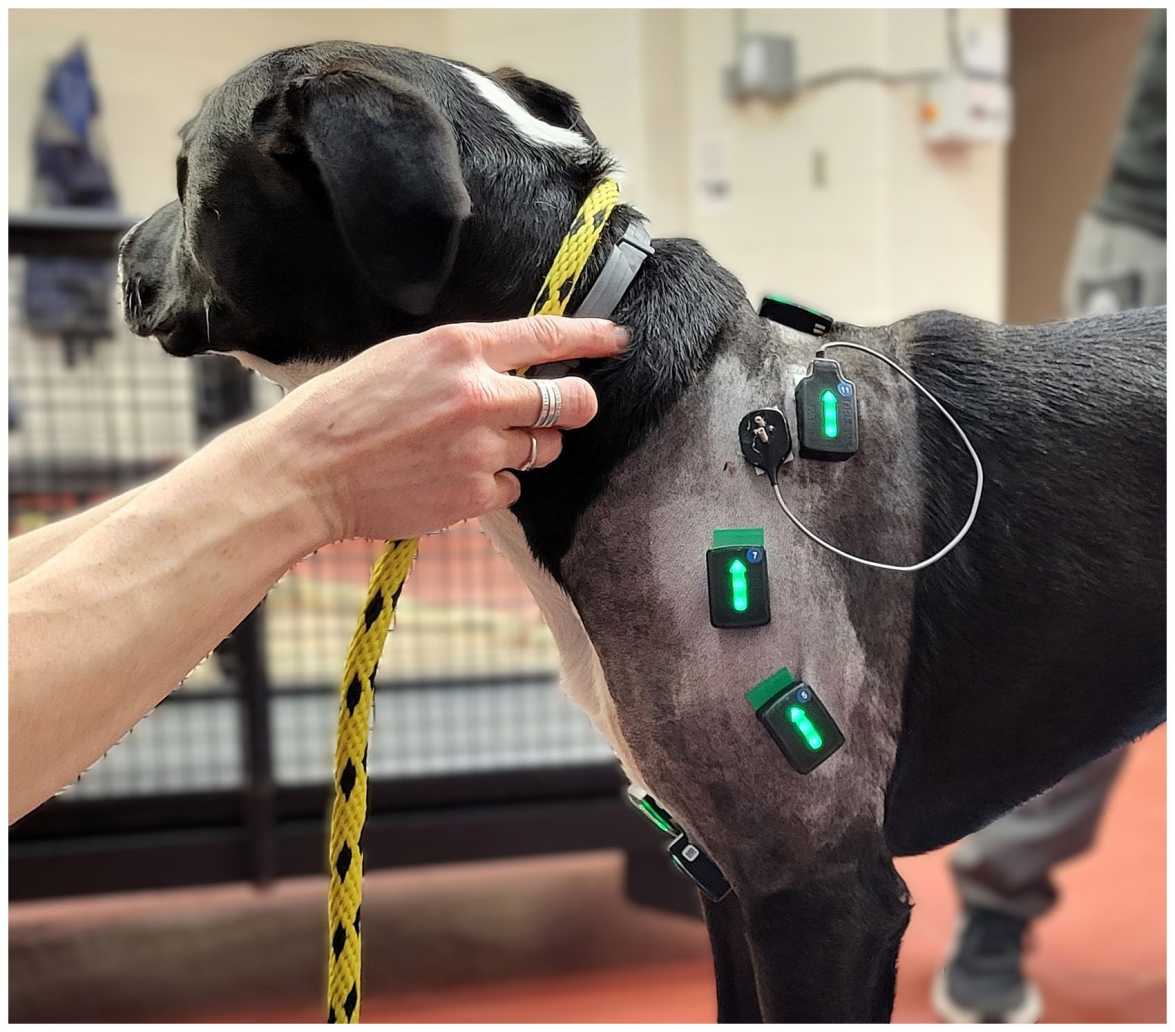
Left lateral view of one study subject showing the experimental set up of the surface and fine wire electromyographic sensors.

Two dogs with behavioral concerns required sedation with IV low dose dexmedetomidine for comfort during instrumentation of the EMG sensors. The remaining dogs were able to undergo sensor placement without sedation. Muscle activity was recorded from six surface EMG Avanti sensors on the skin over the middle of the left and right biceps brachii, deltoideus, and triceps brachii, and two fine wire EMG Avanti sensors (Delsys, Natick, MA, USA) which were placed into the left and right supraspinatus muscles. IMU data were also collected from the right biceps brachii for event detection (Figure 1). Supplemental documents contain the description of all sensor placements. The same two examiners were responsible for sensor placement throughout the study to ensure consistent accurate positioning. Double sided adhesive tape (Trigno Systems adhesive interfaces) and topical adhesive (Loctite Gel Control) were used to fix the sensors to the skin. For fine-wire EMG placement, a subcutaneous area over the supraspinatus site of needle insertion was desensitized with 0.25 mL of 2% lidocaine per side. Caution was taken to remain superficial to prevent alterations in muscle function as previously reported (32). Briefly, a pre-sterilized 27-gauge, 30 mm length needle with pre-loaded paired fine wire electrodes (Rhythmlink, Columbia, SC) was aseptically inserted into the supraspinatus.

### 2.2 Exercises

The treadmill positions were selected and performed for each dog in a random order to remove any order bias. Dogs were asked to walk on a land treadmill at 3.0 km/h in the following positions: level, 5% incline, 10% incline, 5% decline and 10% decline in a randomized order, and all dogs had prior exposure to the treadmill to ensure familiarization (16, 33, 34). Familiarization was different for each dog, but most dogs were habituated after a single training session prior to enrollment in the study. The incline and decline levels of 5 and 10%, respectively, were transitioned with the treadmill moving, and the dog was removed from the treadmill to switch between incline and decline positions. All treadmill positions were recorded during the same test period, and after the dog had acclimatized to each new treadmill position, EMG signals were collected for 30 s and a minimum of 10 gait cycles at a steady walk. Left and right front leg initial contact events were identified from the IMU sensors on the right biceps brachii, and a gait cycle was defined to start at the peak vertical deceleration directly after swing phase which was identified from the sagittal plane gyroscope data.

### 2.3 Data processing

The EMG signals were exported from Trigno Discover (Delsys, Natick, MA, USA) into Visual3d (HAS Motion, Kingston, CA) where they were high pass filtered with a cut off frequency of 40 Hz to reduce movement artifacts. The signals were then full wave rectified, and low pass filtered at 15 Hz to produce an enveloped EMG signal. Peak and average enveloped EMG values were then normalized to the maximum observed signal across all treadmill positions for each muscle separately (35).

### 2.4 Statistical analysis

Average values from the normalized peak and average enveloped EMG signals for each muscle from the 10 gait cycles at the 5 treadmill positions were tested for normality and found suitable for parametric analysis using Shapiro–Wilk tests. Repeated Measures ANOVA tests were performed to explore the effect of the 5 treadmill positions. When significant main effects were seen within a muscle, *post hoc* pairwise comparisons were performed to determine differences between 0, 5% incline, 10% incline, 5% decline, and 10% decline positions using least significant difference tests. All statistical analyses were performed using SPSS version 29 (IBM, USA), and the alpha level was set at (*p* < 0.05).

## 3 Results

Data were collected from twelve dogs and EMG data from nine dogs were deemed acceptable due to signal dropout or technical difficulties. Our participants included 1 German Short-haired Pointer, 1 Wire Haired Pointer, 1 Doberman, 1 German Shepherd, 1 Dutch Shepherd, 1 Golden Retriever, and 6 Mixed Breed dogs between 21.4 and 35.2 kg with body condition scores of 4–6/9, and ages 1–10 years old.

The EMG data from the right side versus left side showed no significant difference allowing for left and right measurements to be pooled. To limit the effect of skin motion artifact on the EMG signal, one author (JR) extensively experienced in analysing EMG data reviewed each trial for each dog to ensure the quality of data included for analysis. Trials with excessive motion artifact was excluded from final analysis. Therefore, of the possible 18 data sets obtained bilaterally from each muscle in 9 dogs, the final data set included 8 EMG observations from the triceps, 7 EMG observations from the biceps, 18 EMG observations from the deltoids and 14 EMG observations from the supraspinatus.

In this study, the peak muscle activity represented the highest amount of muscle activation, and the average muscle activity was an indication of total muscle activation during the gait cycle (32).

### 3.1 Supraspinatus muscle

**Figure 2:**
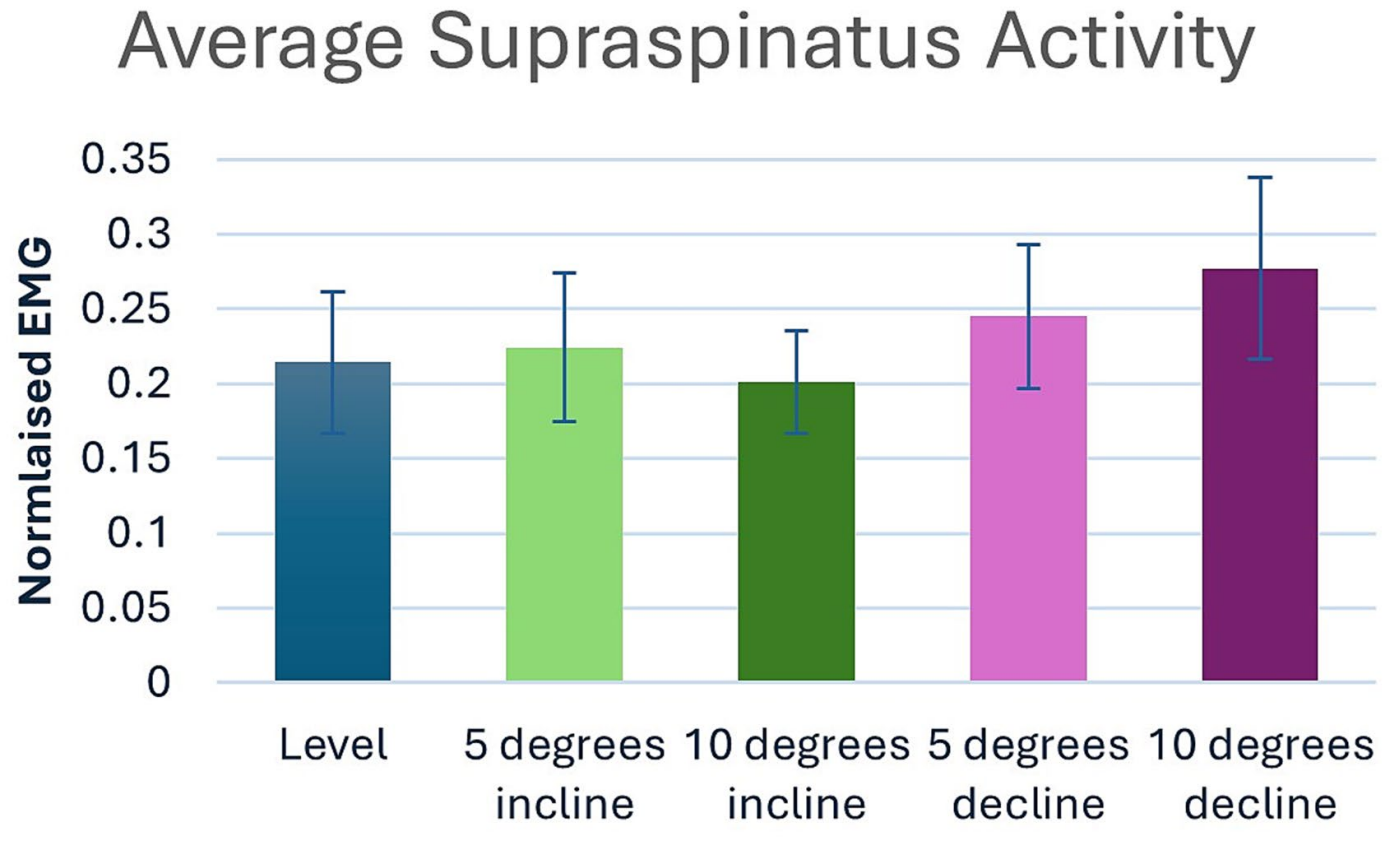
Pairwise comparisons for average supraspinatus electromyography where significant main effects were seen between positions.

Significant main effects were seen for average supraspinatus muscle activity (*p* < 0.001) (Table 1). *Post hoc* pairwise comparisons for average supraspinatus activity showed significant increases between 0 and 5% decline (*p* = 0.019, 14.5%), 0 to 10% decline (*p* < 0.001, 29.4%), 5% decline to 10% decline (*p* < 0.001, 13.0%), and significant decreases between 5% incline and 10% incline (*p* = 0.023, 10.3%), (Tables 2, 3; Figure 2). In addition, significant differences were seen for average supraspinatus activity between various treadmill positions including; 5% incline vs. 10% decline (*p* = 0.004), 10% incline vs. 5% decline (*p* = 0.003), 10% incline vs. 10% decline (*p* < 0.001), (Table 2).

**Table 1:** Mean and standard deviation (sd) for average and peak EMG normalised to the maximum observed signal with main effects (*p* value) and effects sizes (np^2^) between treadmill positions.

	Level	5% Incline	10% Incline	5% Decline	10% Decline	*p* value
	Mean (sd)	Mean (sd)	Mean (sd)	Mean (sd)	Mean (sd)	(np^2^)
Muscle activity						
Peak triceps	0.348 (0.122)	0.387 (0.159)	0.318 (0.147)	0.315 (0.112)	0.378 (0.054)	0.343 (0.14)
Average triceps	0.158 (0.043)	0.177 (0.120)	0.154 (0.087)	0.157 (0.092)	0.167 (0.073)	0.761 (0.06)
Peak biceps	0.337 (0.088)	0.347 (0.080)	0.376 (0.104)	0.314 (0.082)	0.357 (0.078)	0.246 (0.19)
Average biceps	0.179 (0.042)	0.179 (0.038)	0.178 (0.043)	0.185 (0.042)	0.198 (0.037)	0.228 (0.20)
Peak deltoid	0.288 (0.099)	0.299 (0.097)	0.297 (0.010)	0.348 (0.094)	0.366 (0.103)	**0.021** (0.15)
Average deltoid	0.126 (0.030)	0.137 (0.040)	0.131 (0.039)	0.163 (0.050)	0.176 (0.049)	**<0.001** (0.32)
Peak supraspinatus	0.497 (0.090)	0.509 (0.109)	0.489 (0.093)	0.504 (0.113)	0.542 (0.131)	0.057 (0.16)
Average supraspinatus	0.214 (0.047)	0.224 (0.049)	0.201 (0.034)	0.245 (0.048)	0.277 (0.061)	**<0.001** (0.51)

Bold values indicates significant differences *p* <0.05.

**Table 2:** Pairwise comparisons for average and peak EMG where significant main effects were seen between positions.

	Mean difference	*p* value	Lower bound	Upper bound
Maximum deltoid activity				
Level v 5 degrees incline	−0.011	0.681	−0.067	0.045
Level v 10 degrees incline	−0.009	0.727	−0.065	0.047
Level v 5 degrees decline	−0.060	**0.045**	−0.12	−0.001
Level v 10 degrees decline	−0.079	**0.016**	−0.14	−0.017
5 degrees incline v 10 degrees incline	0.002	0.916	−0.031	0.035
5 degrees incline v 5 degrees decline	−0.049	0.111	−0.111	0.013
5 degrees incline v 10 degrees decline	−0.068	0.062	−0.139	0.004
10 degrees incline v 5 degrees decline	−0.051	0.114	−0.116	0.014
10 degrees incline v 10 degrees decline	−0.069	0.068	−0.144	0.006
5 degrees decline v 10 degrees decline	−0.018	0.428	−0.065	0.029
Average deltoid activity				
Level v 5 degrees incline	−0.010	0.291	−0.030	0.010
Level v 10 degrees incline	−0.005	0.540	−0.022	0.012
Level v 5 degrees decline	−0.037	**0.003**	−0.06	−0.014
Level v10 degrees decline	−0.050	**<0.001**	−0.072	−0.027
5 degrees incline v 10 degrees incline	0.005	0.376	−0.007	0.018
5 degrees incline v 5 degrees decline	−0.027	0.055	−0.054	0.001
5 degrees incline v 10 degrees decline	−0.039	**0.009**	−0.067	−0.011
10 degrees incline v 5 degrees decline	−0.032	**0.034**	−0.061	−0.003
10 degrees incline v 10 degrees decline	−0.045	**0.005**	−0.074	−0.016
5 degrees decline v 10 degrees decline	−0.013	0.110	−0.028	0.003
Average supraspinatus activity				
Level v 5 degrees incline	−0.010	0.287	−0.03	0.010
Level v 10 degrees incline	0.013	0.143	−0.005	0.031
Level v 5 degrees decline	−0.031	**0.019**	−0.056	−0.006
Level v10 degrees decline	−0.063	**<0.001**	−0.090	−0.036
5 degrees incline v 10 degrees incline	0.023	**0.023**	0.004	0.042
5 degrees incline v 5 degrees decline	−0.021	0.096	−0.046	0.004
5 degrees incline v 10 degrees decline	−0.053	**0.004**	−0.085	−0.020
10 degrees incline v 5 degrees decline	−0.044	**0.003**	−0.070	−0.018
10 degrees incline v 10 degrees decline	−0.076	**<0.001**	−0.106	−0.046
5 degrees decline v 10 degrees decline	−0.032	**<0.001**	−0.048	−0.016

Bold values indicates significant differences *p* <0.05.

**Table 3:** Percentage differences between positions (negative indicates second value greater than first).

	% Difference					
	Level—5 incline	Level—10 incline	Level—5 decline	Level—10 decline	5 incline—10 incline	5 decline—10 decline
Muscle activity						
Peak triceps	11.23	−8.62	−9.34	8.62	−17.85	19.81
Average triceps	11.87	−2.77	−0.82	5.49	−13.09	6.36
Peak biceps	2.96	11.31	−7.05	5.77	8.11	13.80
Average biceps	0.27	−0.44	3.41	10.57	−0.72	6.92
Peak deltoid	3.85	3.26	**21.02**	**27.32**	−0.56	5.19
Average deltoid	8.22	4.03	**29.35**	**39.24**	−3.87	7.64
Peak supra	2.45	−1.61	1.40	9.22	−3.96	7.70
Average supra	4.71	−6.02	**14.47**	**29.37**	**−10.25**	**13.01**

Bold indicates values of noteworthiness.

### 3.2 Scapular portion of deltoideus muscle

**Figure 3:**
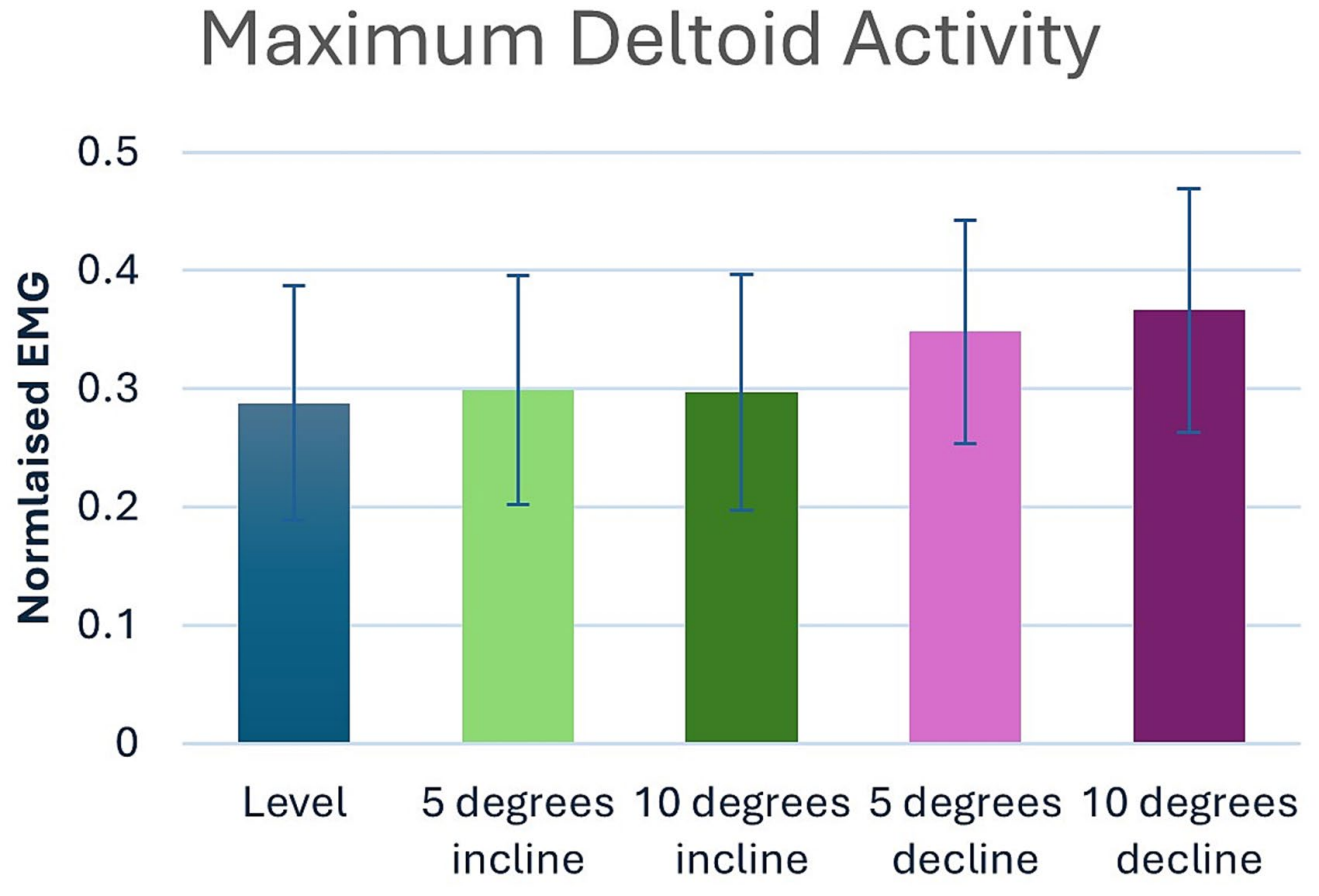
Pairwise comparisons for peak deltoid electromyography where significant main effects were seen between positions.

**Figure 4:**
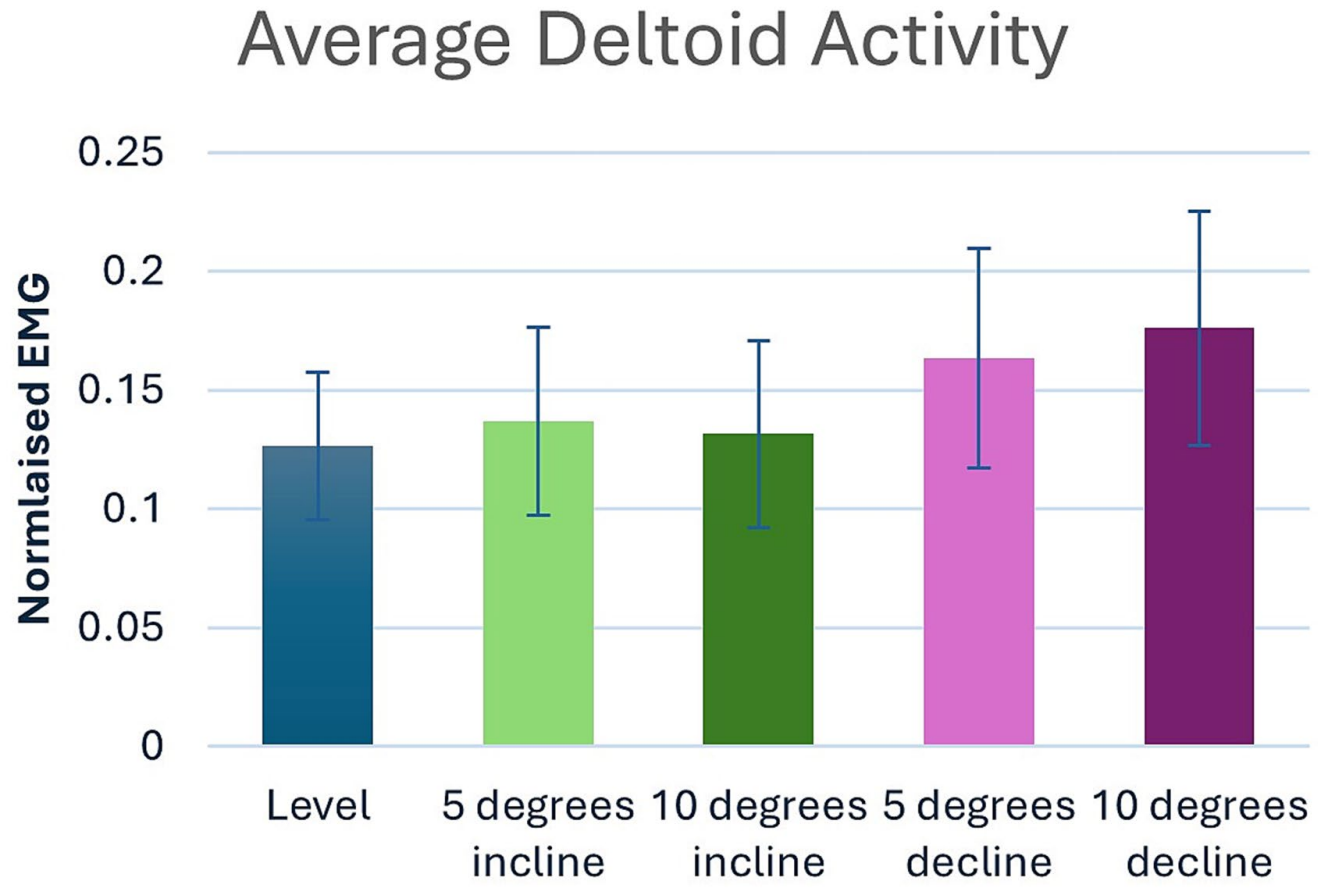
Pairwise comparisons for average deltoid electromyography where significant main effects were seen between positions.

Significant main effects were seen for both average and peak deltoideus muscle activity (*p* = 0.021, *p* < 0.001) respectively (Table 1). *Post hoc* pairwise comparisons for peak deltoideus activity showed significant increases between 0 to 5% decline (*p* = 0.045, 21.0%), and 0 to 10% decline (*p* = 0.016, 27.2%), (Figure 3). For average deltoideus activity significant increases were seen between 0 to 5% decline (*p* = 0.003, 29.4%), and 0 to 10% decline (*p* < 0.001, 39.2%), (Tables 2, 3; Figure 4). In addition, significant differences were seen for average deltoideus activity between various treadmill positions including 5% incline vs. 10% decline (*p* = 0.009), 10% incline vs. 5% decline (*p* = 0.034), 10% incline vs. 10% decline (*p* = 0.005), (Table 2).

### 3.3 Lateral head of triceps brachii muscle

No significant differences were seen for peak or average triceps brachii muscle activity between treadmill positions (Table 1). However similar trends were seen in peak triceps brachii activity when compared to average changes in supraspinatus activity, with a 19.8% increase for the 5–10% decline walking, and a 17.9% decrease for the 5–10% incline walking, and for average triceps brachii activity with a 13.1% decrease for the 5–10% incline walking, and 6.4% increase for the 5–10% decline walking (Table 3).

### 3.4 Biceps brachii muscle

No significant differences were seen for peak or average biceps brachii muscle activity between treadmill positions (Table 1).

## 4 Discussion

Incremental effects of incline and decline treadmill walking have not previously been evaluated, and only a few studies (24–26) have evaluated muscle activity in the canine forelimb. Multiple surveys of sporting dog injuries have reported a higher incidence of injuries to the shoulder, highlighting the need for further studies evaluating the muscular response with common exercises used for rehabilitating shoulder injuries and return to sport conditioning programs (6–8). The supraspinatus and deltoideus were chosen based on their actions of flexion and extension of the shoulder, their role in shoulder joint stabilization, and the frequency of injury to the supraspinatus tendon in dogs (2, 36, 37). The biceps brachii and the triceps brachii were chosen because of their direct involvement with flexion and extension of both the shoulder and elbow, the incidence of biceps tendinopathy, and the triceps brachii being an anti-gravity muscle of the forelimb. The data from this study partially support the hypothesis of increased muscle activity with incremental decline walking, with differences seen in the deltoideus and supraspinatus muscles, but not in the lateral head of the triceps brachii or biceps brachii muscles. We also expected to see decreased muscle activity with incremental incline walking; however, this was only seen in the average supraspinatus muscle activity between the 5 and 10% incline.

### 4.1 Supraspinatus muscle activity and clinical implications

The supraspinatus is active throughout the stance phase, stabilizing the shoulder joint for as long as the limb is load bearing (38). In this study, the supraspinatus showed significant increases in average muscle activity during decline walking. Throughout decline walking, the increased activity of the supraspinatus muscle may be attributed to the braking momentum of the body when going downhill, requiring greater muscle activation with steeper grades. This is consistent with the findings by Cullen et al. (26), who found the highest supraspinatus activation when descending the A-frame. Of particular clinical interest were the incremental changes of the average supraspinatus activity. During decline walking, an additional 13.0% increase in muscle activity was observed at the 10% decline versus the 5% decline position. Conversely, during incline walking, the muscle activity of the supraspinatus decreased by an additional 10.3% at the 10% incline versus the 5% incline position. Direct comparisons of decline walking to incline walking, though statistically significant, did not offer any additional clinical relevance over the direct comparisons to 0%.

These results reveal an ability to increase or decrease supraspinatus muscle activity via incremental changes of decline or incline positions, respectively, during walking. This information may be beneficial when considering the use of treadmill walking for rehabilitation following supraspinatus injuries. For example, a rehabilitation plan for a supraspinatus injury may start initially with walking on a 10% treadmill incline to decrease the supraspinatus activity, and as healing continues, slowly progress to level walking, followed by 5% decline and then 10% decline on a treadmill to systematically increase the load on the muscle. Adjustments to the treadmill position based on these findings may also be useful when designing patient protocols to address muscular strength training, pain and shoulder function during the recovery period.

### 4.2 Scapular portion of deltoideus muscle activity and clinical implications

The scapular portion of the deltoideus muscle is active during the second half of the stance phase where it supports retraction of the limb (38). In this study, the deltoideus muscle activity showed significant increases in both peak and average muscle activity during decline walking, with progressively increased activity as the decline becomes steeper, suggesting a role in braking while going down an incline. Peak deltoideus activity was notable with a 21.0% increase at 5% decline and a 27.2% increase at 10% decline. Average values were remarkable with a 29.4% increase at 5% decline and a 39.2% increase at 10% decline. However, unlike the supraspinatus, no differences were seen within the deltoideus during incline walking. Again, direct comparisons of decline walking to incline walking, though statistically significant, did not offer any additional clinical relevance over the direct comparisons to 0%.

Although the deltoideus does not typically experience direct injury, as the primary flexor of the shoulder and a shared joint stabilizer, it may contribute to shoulder injuries if weak. Decline walking at both the 5 and 10% positions significantly increase the activity of the deltoideus muscle indicating greater peak activation and average muscle activity which could be considered clinically important to increase deltoideus strength. As the forelimb experiences braking forces in a retracted position during decline walking (39), it is plausible the notable increase in both peak and average muscle activity was due to the rise in vertical load and subsequent braking when walking downhill. As dogs redistribute their weight, the moment produced by the forelimb retractors attempts to shift mass onto the hindlimbs and use the shoulders to control the descent despite the increased load (39). During uphill walking, the deltoideus helps to support the shoulder joint, contributing to overall stability and coordination of the body as the dog moves forward and up the incline. However, our data suggests that the deltoideus was not influenced during incline walking at 5 and 10% inclines. This is likely due to the decreased forelimb load and subsequent reduced propulsive forces influencing additional deltoideus muscle contraction during incline walking.

### 4.3 Lateral head of triceps brachii muscle activity and clinical implications

In our study, there were no significant differences for peak or average muscle activity for the lateral head of triceps brachii between treadmill positions. However, the percentage differences between treadmill positions for triceps brachii followed a similar trend as the supraspinatus. The triceps brachii muscle activity increased between the 5% decline and 10% decline. Inversely, the muscle activity decreased between the 5% incline and 10% incline. However, these results were not statistically significant thus these findings should be interpreted with caution when considering clinical implementation.

The lateral head of the triceps brachii muscle is responsible for extension of the elbow joint and is important to counter gravity as well as stabilize the joint (38). It has been assumed that increased forelimb muscle contraction is required during decline walking because the dog braces and brakes when walking downhill, and thus it has been assumed that decline walking may be useful to strengthen the triceps brachii muscles (9). This study did not fully support this hypothesis. Triceps brachii muscle activity while climbing an incline could also be expected to be greater due to the increased elbow extension and subsequent propulsion during incline walking noted in the kinematic study by Carr et al. (40). However, the degrees of incline incorporated in this study was closer to that reported in the kinematic study by Holler et al. (41) which did not show a significant change in elbow extension while walking. Given the Carr et al. (40) study used a 70% grade, the Holler et al. (41) study used an 11% grade, and this study used a 10% grade, this might suggest that steeper incline and decline positions are needed to influence triceps brachii activity.

It is plausible that the smaller number of usable observations for the triceps brachii negatively impacted this analysis. Therefore, a greater sample size is required to explore the effect of incline and decline slopes on triceps brachii muscle activity to confirm that a change from the 0 to 10% decline does not significantly change activity levels. Additional studies evaluating EMG activity of the forelimb musculature are needed to definitively support or refute the results presented in this study with a focus on incline or decline walking and trotting. Future studies should aim to recruit a larger sample size to ensure adequate power. In addition, the consideration of EMG evaluation of the lateral and/or long head of the triceps brachii would allow for a more complete analysis of triceps brachii muscle activity.

### 4.4 Biceps brachii muscle activity and clinical implications

In this study, there were no significant differences for peak or average biceps brachii activity between treadmill positions. As a shoulder extensor and stabilizer, it was anticipated the biceps brachii would experience increased load during decline walking resulting in increased muscle activity. Additionally, as a synergist for the triceps brachii with co-contraction of both muscles during the stance phase of gait (42), it would be assumed the biceps brachii would also experience a positive propulsive effect during incline walking. However, in this study, incline and decline positions of the land treadmill at the walk did not significantly influence biceps brachii muscle activity. This is consistent with the findings from Cullen et al., where biceps brachii muscle activity was lower during descending A-frame (26). Clinically this may indicate that incline and decline walking of 10% or less may not assist in targeting and subsequently strengthening the biceps brachii muscle. This lack of muscle activation may suggest that incline and decline walking need not be excluded from exercise protocols for canine patients with injuries to the biceps brachii as the muscle activity does not appear to be significantly influenced by incline and decline walking.

However, as with the triceps brachii, it is plausible that the smaller number of usable observations for the biceps brachii negatively impacted this analysis. However, *post hoc* sample size calculations from the biceps brachii results, set to achieve 80% power and 5% level of significance, indicated that 271 and 68 EMG observations would be required to indicate a significant difference between 0 and 10% decline walking in peak and average biceps brachii muscle activity, respectively. Again, these large sample sizes likely indicate that biceps brachii muscle activity does not significantly change from the 0 to 10% decline, despite the smaller sample size reported here.

Future research efforts should aim to recruit a larger sample size to ensure an adequate power analysis. Additionally, the consideration of fine-wire EMG over surface EMG for evaluation of the biceps brachii may result in a greater numbers of usable EMG observations.

### 4.5 Limitations

Despite following previously published methods, the signal obtained from the triceps brachii and the biceps brachii muscles did not yield consistently reliable EMG output in all dogs. This resulted in a decreased number of observations which could have affected the power of the analysis particularly on biceps brachii and triceps brachii. In addition the variability in the responses within each of the dogs may have masked the effects of the treadmill positions. Given the large required sample size calculated during *post hoc* power calculations for each muscle indicate there likely is no significant biologic change in muscle activity. Suspected reasons for decreased usable signal include movement artifact due to skin displacement. Additionally, muscle activity while walking on a treadmill may not be directly comparable to muscle activity during over ground walking. However, rehabilitation programs are commonly performed on treadmills making the findings of this study clinically relevant.

## 5 Conclusion

Given the prevalence and significance of canine shoulder injuries, specifically supraspinatus and biceps tendinopathies, this study showed implementing a 5% incline, 10% incline, 5% decline or 10% decline during walking could significantly impact shoulder muscle activity and therefore be used with confidence in customized progressive rehabilitation and conditioning programs. This study supports the use of decline walking to activate the supraspinatus and deltoideus muscles and provides new insights regarding the muscle activity of the thoracic limbs during various treadmill positions at the walk. Average EMG activity of the supraspinatus muscle significantly increased during decline walking and decreased during incline walking. The impact of incremental treadmill positions on the average supraspinatus activity, along with increases in the average and peak EMG activity of the deltoideus muscle during decline walking should be considered when developing a therapeutic exercise plan in canine patients with shoulder injuries.

## Data Availability

The raw data supporting the conclusions of this article will be made available by the authors, without undue reservation.
