# Supplementary material for: Forelimb muscle activity during level and progressive incline and decline walking in dogs and implications for rehabilitation
Source: Front Vet Sci. 2025 Aug 29;12:1649009. doi: 10.3389/fvets.2025.1649009 (PMC12425894; doi:10.3389/fvets.2025.1649009)
Supplement: Supplementary file 1 [file Data_Sheet_1.pdf]

Supplemental Documents:

Location of surface EMG sensor placement:

Biceps Brachii (BB): sensors were placed parallel to and on a line from the cranial aspect of the greater tubercle of the humerus, mid-way between the point where the pectoralis/brachiocephalicus crosses over it and where it inserts on the elbow (Garcia, 2014)

Triceps Brachii (TB): sensors were placed parallel to and on a line mid-way between the lateral aspect of the greater tubercle of the humerus to the proximal-most aspect of the olecranon of the ulna (Garcia, 2014)

Deltoideus (DT): sensors were placed parallel to and on a line mid-way from the deltoid tuberosity of the humerus to the scapular spine

Location of fine wire EMG sensor placement:

Wire placement:

Supraspinatus (SS): 1) shoulder in neutral 2) locate cranial border of scapula 3) needle insertion 2 cm proximal to the cranial border of the scapula and needle directed proximally into the central muscle belly distance: 2 cm depth: 1.5 cm (Cullen 2015)

Reference Contact Sensor placement:

Supraspinatus (SS): sensors were placed around the area of the caudal scapula

Spring adapter placement:

Supraspinatus (SS): adapters were placed just proximal, caudal, or distal to the fine wire insertion area pending the size of the dog, taking care to not interfere with the wires as they

Location of the IMU sensor placement:

Cranial thoracic IMU: sensor was placed at the base of the neck, between the cranial angles of the scapulas and centered over midline

Lumbosacral IMU: sensor was placed between the wings of the ilium and centered over midline

Right Biceps IMU: see location of Biceps Brachii (BB) surface sensor placement
